# Discovery of novel quinoline-based analogues of combretastatin A-4 as tubulin polymerisation inhibitors with apoptosis inducing activity and potent anticancer effect

**DOI:** 10.1080/14756366.2021.1899168

**Published:** 2021-03-18

**Authors:** Tarek S. Ibrahim, Mohamed M. Hawwas, Azizah M. Malebari, Ehab S. Taher, Abdelsattar M. Omar, Thikryat Neamatallah, Zakaria K. Abdel-Samii, Martin K. Safo, Yaseen A. M. M. Elshaier

**Affiliations:** a Department of Pharmaceutical Chemistry, Faculty of Pharmacy, King Abdulaziz University, Jeddah, Saudi Arabia; b Department of Pharmaceutical Organic Chemistry, Faculty of Pharmacy, Zagazig University, Zagazig, Egypt; c Department of Pharmaceutical Organic Chemistry, Faculty of Pharmacy, Al-Azhar University, Assiut, Egypt; d Department of Pharmaceutical Chemistry, Faculty of Pharmacy, Al-Azhar University, Cairo, Egypt; e Department of Pharmacology and toxicology, Faculty of Pharmacy, King Abdulaziz University, Jeddah, Saudi Arabia; f Institute for Structural Biology, Drug Discovery and Development, Department of Medicinal Chemistry, School of Pharmacy, Virginia Commonwealth University, Richmond, VA, USA; g Department of Organic and Medicinal Chemistry, Faculty of Pharmacy, University of Sadat City, Menoufia, Egypt

**Keywords:** Combretastatin A-4, quinoline, apoptosis

## Abstract

A new series of quinoline derivatives of combretastatin A-4 have been designed, synthesised and demonstrated as tubulin polymerisation inhibitors. These novel compounds showed significant antiproliferative activities, among them, **12c** exhibited the most potent inhibitory activity against different cancer cell lines (MCF-7, HL-60, HCT-116 and HeLa) with IC_50_ ranging from 0.010 to 0.042 µM, and with selectivity profile against MCF-10A non-cancer cells. Further mechanistic studies suggest that **12c** can inhibit tubulin polymerisation and cell migration, leading to G_2_/M phase arrest. Besides, **12c** induces apoptosis *via* a mitochondrial-dependant apoptosis pathway and caused reactive oxygen stress generation in MCF-7 cells. These results provide guidance for further rational development of potent tubulin polymerisation inhibitors for the treatment of cancer.

**Highlights**
- A novel series of quinoline derivatives of combretastatin A-4 have been designed and synthesised.
- Compound **12c** showed significant antiproliferative activities against different cancer cell lines.
- Compound **12c** effectively inhibited tubulin polymerisation and competed with [^3^H] colchicine in binding to tubulin.
- Compound **12c** arrested the cell cycle at G_2_/M phase, effectively inducing apoptosis and inhibition of cell migration.

## Graphical Abstract

**Figure:**
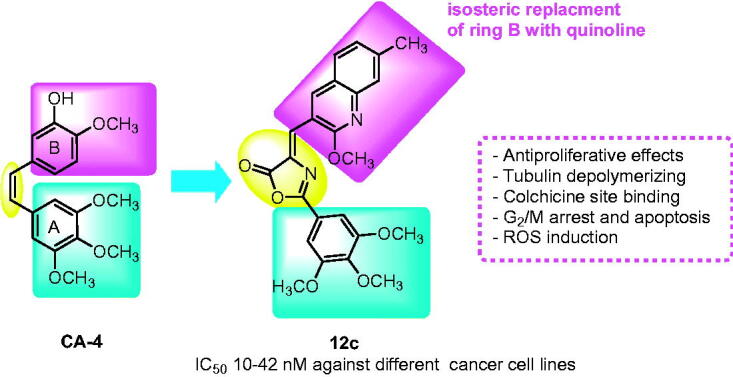

## 1. Introduction

Cancer is a disease of an uncontrolled growth and abnormal division of cells, which leads to death. In recent years, targeted antineoplastic agents have become an effective treatment choice for cancer^1^, with pharmaceutical companies focussing on targeted therapies against different and special cancer types^2^. Tubulin polymerisation inhibitors represent one of the most well-known and potential examples of such targeted cancer therapies^3^. Tubulin is a globular protein that performs a substantial function in cell mitosis. Microtubules (MTs), which represent the basic constituents of eukaryotic cell, are cytoskeletons constructed by the association of α- and β-tubulin heterodimers with a head and tail pattern to form hollow cylindrical tubes (nearly 25 nm in diameter)^4–7^. MTs play a crucial role in many fundamental cellular activities, such as motility, cell formation, cell secretion, signalling, maintenance of cell shape, regulation of intracellular transport and cell division^8–10^. Due to these multiple functions, microtubule system has become an attractive target for cancer chemotherapy^11^^,^^12^. Disruption of MTs or tubulin dynamics exposes the cell to mitotic arrest of the cell cycle at G_2_/M phase, and consequently induction of cellular apoptosis^13^^,^^14^. Several microtubule-interfering agents (MIAs) have been identified, e.g. paclitaxel, vincristine, and colchicine that are obtained from the natural products, taxol, vinca and colchicine, respectively. MIAs are known to bind to tubulin at specific binding sites that are classified as taxol, vinca and colchicine sites to either enhance or inhibit tubulin polymerisation.^15^ For example, microtubule stabilisers, e.g. paclitaxel stimulate microtubule polymerisation^16^, while microtubule destabilizers, e.g. colchicine, and the vinca alkaloids vinblastine and vincristine inhibit polymerisation of microtubules^17^.

**Figure 1.**
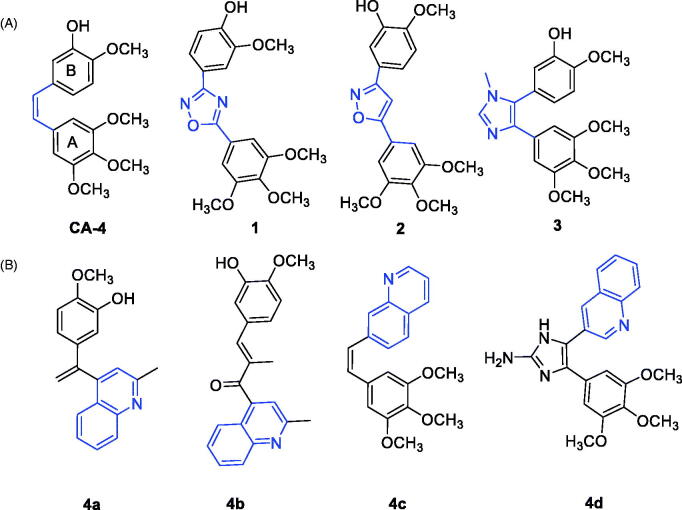
(A) Chemical structure of CA-4 and its analogues; (B) tubulin targeting agents bearing quinoline moiety.

Significant attention is now focussed on colchicine binding site inhibitors due to their positive impact on ABC-transporter-mediated drug resistance^18^^,^^19^. Combretastatin A-4 (CA-4), (**1**, Figure 1) has been reported as the most potent antimitotic agent of this family against several tumour cells^20^. CA-4 was first isolated from the bark of the willow tree *Combretum caffrum* from South Africa in 1989^21^. CA-4 has a vascular disrupting activity against tumour cell vasculature by preventing blood supply to solid tumour, resulting in apoptosis^22–24^. Given its structural simplicity, CA-4 has been studied as a lead pharmacophore for deciphering tubulin functions and properties^25^. Phases II and III clinical studies are currently ongoing with tubulin-targeted drugs^24^^,^^26^. Structure activity relationships (SAR) studies with CA-4 have revealed three important structural features (Figure 1). These include: (i) a 3,4,5-trimethoxy moiety on ring A that is essential for activity; (ii) a *cis*-configuration of both aromatic rings that is essential for activity (*trans*-orientation is inactive); (iii) the presence of small substituent on ring B, e.g. methoxy group that is important for activity. The *cis*-alkene configuration in CA-4 allows the aromatic rings to assume optimal binding orientation for interactions with the colchicine binding site. Unfortunately, the *cis* configuration of CA-4 has a propensity for undergoing transformation to the inactive *trans* configuration upon storage and during *in vivo* metabolism. To overcome this, many structural modifications of CA-4 have been undertaken where the *cis* double bond is replaced with heterocycles, either monocyclic, such as oxadiazole, isoxazole and imidazole, resulting in compounds, such as **1**, **2** and **3** respectively (Figure 1)^27–33^ or fused heterocyclic, such as pyrazolopyridines^34^, triazolopyridines^35^ and triazolothiadiazine derivatives^36^. These compounds, like CA-4 showed pronounced activity against a panel of cancer cell lines.

Quinoline derivatives are popular for the treatment of malaria^37^^,^^38^. Moreover, quinoline heterocyclic containing compounds demonstrate potent anticancer activities with different modes of actions, including inhibition of proteasome, tyrosine kinases, and tubulin polymerisation^39–41^. Previous studies have reported the antiproliferative activity of CA-4, isoCA-4 or chalcone compounds containing quinoline scaffold, either as ring A bioisoster, e.g. **4a**^23^ and **4b**^42^ or ring B bioisoster, e.g. **4c**^43–45^ and **4d**^46^. These compounds demonstrate the potential of the quinoline ring as a template for developing more promising tubulin polymerisation inhibitors and antiproliferative agents.

**Figure 2.**
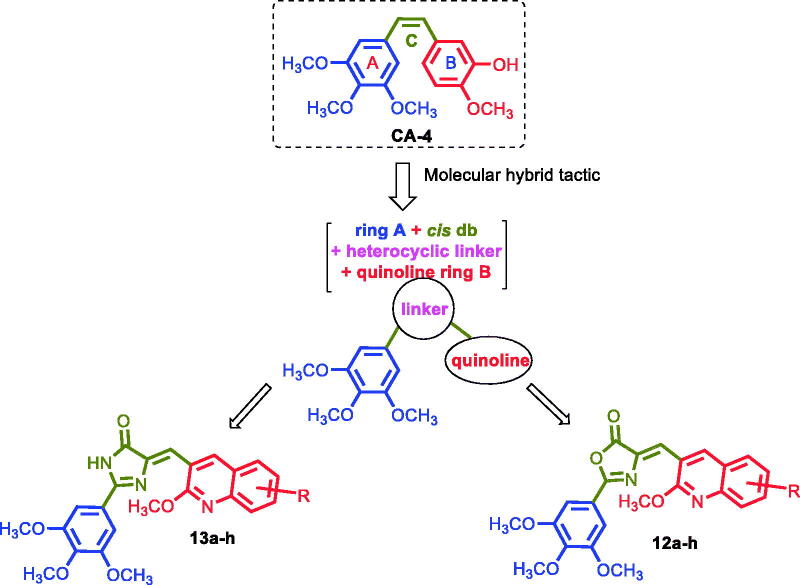
Structure of CA-4 and our rationalised compounds.

In this work, we optimised CA-4 into a series of novel hybrid quinoline derivatives as potent tubulin inhibitor, which involves introducing a rigid oxazolone or imidazolone between rings A and B to maintain the cis configuration, as well as targeting the quinolyl moiety (ring B), by varying the electronic substituents effect while maintaining the 3,4,5-trimethoxyphenyl moiety as present in ring A of CA-4 (Figure 2). Following, we synthesised several analogues that constitute two classes of compounds: the oxazolones (Compounds **12a–h)** and the imidazolones **(**Compounds **13a–h**). The compounds have been screened for their antiproliferative activities against a variety of cancer cell lines, as well as studied for their mechanism of action. We expect the results to lead to better understanding of the mechanistic mode of the compounds’ activity against tubulin and provide guidance for further development of potent anticancer drugs.

## 2. Experimental section

### 2.1. Chemistry

Melting points were determined with a Gallenkamp (London, UK) melting point apparatus and are uncorrected. IR spectra (KBr, cm^−1^) were recorded on Bruker Vector, 22FT-IR [Fourier Transform Infra-red (FTIR), Germany] spectrometer. Unless otherwise specified, proton (^1^H) and carbon (^13^C) NMR spectra were recorded at room temperature in base filtered CDCl_3_ on a spectrometer operating at 400 & 300 MHz for proton and 100 & 75 MHz for carbon nuclei. The signal due to residual CHCl_3_ appearing at δ H 7.26 and (CH_3_)_2_SO appearing at δ H 2.5 and the central resonance of the CDCl_3_ “triplet” appearing at δ C 77.0 and for (CD_3_)_2_SO “multiplet” appearing at δ C 39.0 were used to reference 1H and ^13^C NMR spectra, respectively. ^1^H NMR data are recorded as follows: chemical shift (δ) [multiplicity, coupling constant(s) J (Hz), relative integral] where multiplicity is defined as s = singlet; d = doublet; t = triplet; q = quartette; and m = multiplet or combinations of the above. Elemental analyses were determined using Manual Elemental Analyser Heraeus (Germany) and Automatic Elemental Analyser CHN Model 2400 Perkin Elmer (Waltham, MA, USA) at Microanalytical Centre, Faculty of Science, Cairo University, Egypt. All the elemental analyses results corresponded to calculated values within experimental error. Progress of reactions was monitored by thin-layer chromatography (TLC) using precoated TLC sheets with Ultraviolet (UV) fluorescent silica gel (Merck 60F254), and spots were visualised by iodine vapours or irradiation with UV light (254 nm). All chemicals were purchased from Sigma-Aldrich or Lancaster Synthesis Corporation (UK). Intermediates **6–8a–i** were prepared according to reported procedure^47^^,^^48^.

#### 2.1.1. General procedure for preparation of oxazolones (12a–h)

A mixture of *N*-(3,4,5-trimethoxybenzoyl)glycine (0.30 g, 1.10 mmol) and the appropriate aldehydes **8a–h** (1.00 mmol) in acetic anhydride (1 ml) and fused sodium acetate (0.1 g, 1.2 mmol) was heated on an oil bath at 80 °C for 2 h. After cooling down at room temperature the mixture was allowed to stand for 24 h at 0 °C. The precipitate was filtered off and washed three times with ice-cooled ethanol (10 ml), and the product crystallised from ethanol.

##### 2.1.1.1. 4-[(2-Methoxyquinolin-3-yl)methylene]-2–(3,4,5-trimethoxyphenyl)oxazol-5(4H)-one (12a)

Yellow solid, Yield (81%); m.p. 215–217 °C. IR (KBr): υ = 1776 (C=O), 1621 (C=N), 1599 (C=C) cm^−1^. ^1^H NMR (400 MHz, CDCl_3_) δ: 9.48 (s, 1H, Ar-H), 7.86–7.81 (m, 2H, Ar-H), 7.71–7.67 (m, 2H, Ar-H), 7.45–7.41 (m, 3H, Ar-H), 4.15 (s, 3H, OCH_3_), 4.00 (s, 9H, 3 OCH_3_) ppm. ^13^C NMR (100 MHz, CDCl_3_) δ: 167.1, 163.9, 160.0, 153.5, 147.1, 143.1, 142.4, 134.6, 131.5, 128.9, 127.1, 125.2, 124.7, 123.6, 120.1, 118.5, 105.8, 61.1, 56.4, 54.1 ppm. MS (70 eV): *m/z* (%): 420 (7.79) [M^+^]; Anal. Calcd for C_23_H_20_N_2_O_6_: C, 65.71; H, 4.80; N, 6.66. Found: C, 65.64; H, 4.74; N, 6.71

##### 2.1.1.2. 4-[(2-Methoxy-6-methylquinolin-3-yl)methylene]-2–(3,4,5-trimethoxyphenyl) oxazol-5(4H)-one (12b)

Pale yellow solid, Yield (79%); m.p. 218–220 °C. ^1^H NMR (400 MHz, CDCl_3_) δ: 9.36 (s, 1H, Ar-H), 7.72 (t, *J* = 4 Hz, 2H, Ar-H), 7.56 (s, 1H, Ar-H), 7.49 (d, *J* = 8.5 Hz, 1H, Ar-H), 7.41 (s, 2H, Ar-H), 4.11 (s, 3H, OCH_3_), 3.98 (d, *J* = 8.0 Hz, 9H, 3OCH_3_) and 2.51 (s, 3H, CH_3_) ppm. ^13^C NMR (100 MHz, CDCl_3_) δ: 167.2, 163.8, 159.7, 153.5, 145.7, 143.1, 141.8, 134.4, 134.3, 133.6, 127.8, 126.9, 125.1, 124.1, 120.3, 118.1, 110.0, 105.7, 61.1, 56.4, 53.9, 21.3 ppm. MS (70 eV): *m/z* (%): 434 (9.79) [M^+^]; Anal. Calcd for C_24_H_22_N_2_O_6_: C, 66.35; H, 5.10; N, 6.45. Found: C, 66.29; H, 5.04; N, 6.50.

##### 2.1.1.3. 4-[(2-Methoxy-7-methylquinolin-3-yl)methylene]-2–(3,4,5-trimethoxyphenyl) oxazol-5(4H)-one (12c)

Yellow solid, Yield (84%); m.p. 233–235 °C. ^1^H NMR (400 MHz, CDCl_3_) δ: 9.50 (s, 1H, Ar-H), 7.76–7.73 (m, 3H, Ar-H),7.45 (s, 2H, Ar-H), 7.30–7.28 (m, 1H, Ar-H), 4.20 (s, 3H, OCH_3_), 4.00 (d, *J* = 8.0 Hz, 9H, 3 OCH_3_), 2.58 (s, 3H, CH_3_) ppm. ^13^C NMR (100 MHz, CDCl_3_) δ: 167.2, 163.8, 162.6, 160.2, 153.5, 143.1, 142.8, 142.5, 134.3, 128.7, 127.0, 126.3, 123.9, 123.1, 120.2, 117.6, 105.7, 61.1, 56.4, 54.6, 22.1 ppm. MS (70 eV): *m/z* (%): 434 (6.90) [M^+^]; Anal. Calcd for C_24_H_22_N_2_O_6_: C, 66.35; H, 5.10; N, 6.45. Found: C, 66.32; H, 5.05; N, 6.47.

##### 2.1.1.4. 4-[(2-Methoxy-8-methylquinolin-3-yl)methylene]-2–(3,4,5-trimethoxyphenyl) oxazol-5(4H)-one (12d)

Yellow solid, Yield (82%); m.p. 236–238 °C. ^1^H NMR (400 MHz, CDCl_3_) δ: 9.44 (s, 1H, Ar-H), 7.73 (s, 1H, Ar-H), 7.64 (d, *J* = 8.0 Hz, 1H, Ar-H), 7.52 (d, *J* = 6.40 Hz, 1H, Ar-H), 7.39 (s, 2H, Ar-H), 7.32–7.28 (m, 1H, Ar-H), 4.12 (s, 3H, OCH_3_), 3.98 (s, 9H, 3 OCH_3_), 2.68 (s, 3H, CH_3_) ppm. ^13^C NMR (100 MHz, CDCl_3_) δ: 167.2, 163.8, 159.1, 153.7, 146.0, 143.1, 142.7, 135.4, 134.3, 131.6, 126.7, 125.0, 124.3, 124.0, 120.2, 118.0, 105.6, 61.1, 56.3, 53.7, 17.5 ppm. MS (70 eV): *m/z* (%): 434 (9.50) [M^+^]; Anal. Calcd for C_24_H_22_N_2_O_6_: C, 66.35; H, 5.10; N, 6.45. Found: C, 66.30; H, 5.04; N, 6.51.

##### 2.1.1.5. 4-[2,6-Dimethoxyquinolin-3-yl)methylene]-2–(3,4,5-trimethoxyphenyl)oxazol-5(4H)-one (12e)

Yellow solid, Yield (84%); m.p. 242–244 °C. 1784 (C=O), 1618 (C=N), 1586 (C=C) cm^−1^. ^1^H NMR (400 MHz, CDCl_3_) δ: 9.33 (s, 1H, Ar-H), 7.73 (d, *J* = 8.0 Hz, 2H, Ar-H), 7.42–7.32 (m, 3H, Ar-H), 7.12 (s, 1H, Ar-H), 4.10 (s, 3H, OCH_3_), 3.99 (s, 9H, 3 OCH_3_) 3.93 (s, 3H, OCH_3_) ppm. ^13^C NMR (100 MHz, CDCl_3_) δ: 167.1, 163.8, 158.9, 156.4, 153.5, 143.2, 143.0, 141.1, 134.5, 128.5, 125.7, 124.2, 123.0, 120.2, 118.4, 107.4, 105.9, 61.2, 56.5, 55.6, 53.8 ppm. MS (70 eV): *m/z* (%): 450 (8.40) [M^+^]; Anal. Calcd for C_24_H_22_N_2_O_7_: C, 64.00; H, 4.92; N, 6.22. Found: C, 63.94; H, 4.87; N, 6.28.

##### 2.1.1.6. 4-[(2,7-Dimethoxyquinolin-3-yl)methylene]-2–(3,4,5-trimethoxyphenyl)oxazol-5(4H)-one (12f)

Yellow solid, Yield (84%); m.p. 221–223 °C. ^1^H NMR (400 MHz, CDCl_3_) δ: 9.39 (s, 1H, Ar-H), 7.70–7.66 (m, 2H, Ar-H), 7.38 (s, 2H, Ar-H), 7.16 (s, 1H, Ar-H), 7.04 (d, *J* = 8.0 Hz, 1H, Ar-H), 4.11 (s, 3H, OCH_3_), 3.97 (s, 9H, 3OCH_3_) 3.95 (s, 3H, OCH_3_) ppm. ^13^C NMR (100 MHz, CDCl_3_) δ: 167.4, 163.3, 162.8, 160.7, 153.5, 149.7, 142.8, 142.0, 133.3, 130.2, 124.4, 120.4, 120.1, 117.2, 115.9, 106.4, 105.5, 61.2, 56.2, 55.6, 53.9 ppm. MS (70 eV): *m/z* (%): 450 (7.30) [M^+^]; Anal. Calcd for C_24_H_22_N_2_O_7_: C, 64.00; H, 4.92; N, 6.22. Found: C, 63.96; H, 4.96; N, 6.25.

##### 2.1.1.7. 4-[(7-Isopropoxy-2-methoxyquinolin-3-yl)methylene]-2–(3,4,5-trimethoxy phenyl)oxazol-5(4H)-one (12g)

Yellow solid, Yield (77%); m.p. 227–229 °C. ^1^H NMR (400 MHz, CDCl_3_) δ ppm: 9.41 (s, 1H, Ar-H), 7.74–7.67 (m, 2H, Ar-H), 7.41 (s, 2H, Ar-H),7.16–7.00 (m, 2H, Ar-H), 4.87–4.76 (m, 1H, OCH-), 4.12 (s, 3H, OCH_3_), 3.98 (s, 9H, 3 OCH_3_), 1.45 (d, *J* = 4.0 Hz, 6H, 2 CH_3_) ppm. ^13^C NMR (100 MHz, CDCl_3_) δ: 167.4, 163.1, 161.2, 160.7, 153.5, 149.5, 142.9, 141.9, 133.2, 130.3, 124.6, 120.4, 119.9, 118.2, 115.7, 107.9, 105.5, 70.3, 61.3, 56.4, 53.9, 22.0 ppm. MS (70 eV): *m/z* (%): 478 (9.50) [M^+^]; Anal. Calcd for C_26_H_26_N_2_O_7_: C, 65.26; H, 5.48; N, 5.85. Found: C, 65.21; H, 5.45; N, 5.91.

##### 2.1.1.8. 4-[(7-(Benzyloxy)-2-methoxyquinolin-3-yl)methylene]-2–(3,4,5-trimethoxy phenyl)oxazol-5(4H)-one (12h)

Pale yellow solid, Yield (73%); m.p. 247–249 °C. ^1^H NMR (400 MHz, CDCl_3_) δ: 9.43 (s, 1H, Ar-H), 7.72 (t, *J* = 8 Hz, 2H, Ar-H), 7.51–7.35 (m, 6H, Ar-H), 7.28–7.26 (m, 2H, Ar-H), 7.14 (d, 1H, *J* = 8.0 Hz, 1H, Ar-H), 5.21 (s, 2H, OCH_2_−), 4.12 (s, 3H, OCH_3_), 3.98 (s, 9H, 3 OCH_3_) ppm. ^13^C NMR (100 MHz, CDCl_3_) δ: 167.4, 163.3, 161.9, 160.8, 153.5, 149.4, 142.9, 141.9, 136.2, 133.5, 130.2, 128.7, 128.3, 127.7, 124.4, 120.4, 120.3, 117.5, 116.0, 107.5, 105.6, 70.3, 61.3, 56.4, 53.9 ppm. MS (70 eV): *m/z* (%): 526 (7.30) [M^+^]; Anal. Calcd for C_30_H_26_N_2_O_7_: C, 68.43; H, 4.98; N, 5.35. Found: C, 68.39; H, 4.92; N, 5.41.

#### 2.1.2. *General procedure for preparation of* (13a–h)

The appropriate oxazolones **12a–h** (1 mmol) was stirred and heated under reflux in ethanol (10 ml) containing ammonium hydroxide (10 ml), and the reaction monitored by TLC. After completion of the reaction in 24 h, the solvent was concentrated and cooled, and the precipitate was filtered off and crystallised from ethanol.

##### 2.1.2.1. 5-[(2-Methoxyquinolin-3-yl)methylene]-2–(3,4,5-trimethoxyphenyl)-3,5-dihydro-4H-imidazol-4-one (13a)

Yellow solid, Yield (81%); m.p. 230–232 °C. IR (KBr): υ = 3222 (NH), 1709 (C=O), 1642 (C=N), 1615, 1589 (C=C) cm^−1^. ^1^H NMR (400 MHz, DMSO-d_6_) δ: 12.22 (s, 1H, exch., NH), 9.69 (s, 1H, Ar-H), 8.03 (d, *J* = 8.0 Hz, 1H, Ar-H), 7.80–7.70 (m, 2H, Ar-H), 7.59 (s, 2H, Ar-H), 7.49 (t, *J* = 8.0 Hz, 1H, Ar-H), 7.28 (s, 1H, Ar-H), 4.11 (s, 3H, OCH_3_), 3.94 (s, 6H, 2OCH_3_), 3.80 (s, 3H, OCH_3_) ppm. ^13^C NMR (100 MHz, DMSO-d_6_) δ: 187.4, 165.0, 164.2, 161.3, 157.9, 153.5, 146.7, 140.9, 132.2, 128.9, 126.7, 125.2, 123.9, 118.4, 108.7, 104.2, 60.3, 56.3, 54.1 ppm. MS (70 eV): *m/z* (%): 419 (5.40) [M^+^]; Anal. Calcd for C_23_H_21_N_3_O_5_: C, 65.86; H, 5.05; N, 10.02. Found: C, 65.81; H, 4.99; N, 10.09.

##### 2.1.2.2. 5-[(2-Methoxy-6-methylquinolin-3-yl)methylene]-2–(3,4,5-trimethoxyphenyl)-3,5-dihydro-4H-imidazol-4-one (13b)

Yellow solid, Yield (81%); m.p. 223–225 °C. ^1^HNMR (400 MHz, DMSO-d_6_) δ: 12.18 (s, 1H, exch., NH), 9.55 (s, 1H, Ar-H), 7.74–7.54 (m, 5H, Ar-H), 7.26–7.24 (d, *J* = 6.6 Hz, 1H, Ar-H), 4.07 (s, 3H, OCH_3_), 3.93 (s, 6H, OCH_3_), 3.80 (s, 3H, OCH_3_), 2.47 (s, 3H, CH_3_) ppm. ^13^C NMR (100 MHz, DMSO-d_6_) δ: 172.0, 162.2, 156.0, 153.7, 144.6, 142.6, 142.4, 141.2, 134.4, 133.4, 128.1, 126.8, 125.4, 123.2, 119.3, 116.2, 110.0, 106.2, 60.7, 56.8, 54.2, 21.3 ppm. MS (70 eV): *m/z* (%): 433 (5.60) [M^+^]; Anal. Calcd for C_24_H_23_N_3_O_5_: C, 66.50; H, 5.35; N, 9.69. Found: C, 66.41; H, 5.28; N, 9.75.

##### 2.1.2.3. 5-[(2-Methoxy-7-methylquinolin-3-yl)methylene]-2–(3,4,5-trimethoxyphenyl)-3,5-dihydro-4H-imidazol-4-one (13c)

Yellow solid, Yield (84%); m.p. 236–238 °C. IR (KBr): υ = 3215 (NH), 1711 (C=O), 1639 (C=N), 1590 (C=C) cm^−1^. ^1^H NMR (400 MHz, DMSO-d_6_) δ: 12.17 (s, 1H, exch. NH), 9.63 (s, 1H, Ar-H), 7.92 (d, *J* = 8.0 Hz, 1H, Ar-H), 7.58 (s, 3H, Ar-H), 7.32 (d, *J* = 8 Hz, 1H, Ar-H), 7.27 (s, 1H, Ar-H), 4.09 (s, 3H, OCH_3_), 3.94 (s, 6H, 2OCH_3_), 3.80 (s, 3H, OCH_3_), 2.53 (s, 3H, CH_3_) ppm. ^13^C NMR (100 MHz, DMSO-d_6_) δ: 162.6, 162.5, 160.3, 153.9, 142.2, 141.5, 129.1, 127.1, 126.3, 123.6, 118.3, 115.8, 105.6, 60.8, 56.5, 54.2, 22.0 ppm. MS (70 eV): *m/z* (%): 433 (8.40) [M^+^]; Anal. Calcd for C_24_H_23_N_3_O_5_: C, 66.50; H, 5.35; N, 9.69. Found: C, 66.44; H, 5.32; N, 9.72.

##### 2.1.2.4. 5-[(2-Methoxy-8-methylquinolin-3-yl)methylene]-2–(3,4,5-trimethoxy phenyl)-3,5-dihydro-4H-imidazol-4-one (13d)

Yellow solid, Yield (82%); m.p. 233–235 °C. ^1^H NMR (400 MHz, DMSO-d_6_) δ: 12.16 (s, 1H, exch., NH), 9.59 (s, 1H, Ar-H), 7.80 (s, 1H, Ar-H), 7.70 (d, *J* = 8 Hz, 1H, Ar-H), 7.59–7.56 (m, 3H, Ar-H), 7.29 (s, 1H, Ar-H), 4.10 (s, 3H, OCH_3_), 3.95 (s, 6H, 2OCH_3_), 3.80 (s, 3H, OCH_3_), 2.49 (s, 3H, CH_3_) ppm. ^13^C NMR (100 MHz, DMSO-d_6_) δ: 161.9, 158.2, 153.3, 142.2, 141.6, 141.5, 134.2, 131.3, 126.7, 124.8, 124.5, 118.6, 115.4, 105.3, 60.2, 56.1, 53.5, 17.1 ppm. MS (70 eV): *m/z* (%): 433 (7.12) [M^+^]; Anal. Calcd for C_24_H_23_N_3_O_5_: C, 66.50; H, 5.35; N, 9.69. Found: C, 66.47; H, 5.30; N, 9.73.

##### 2.1.2.5. 5-[(2,6-Dimethoxyquinolin-3-yl)methylene]-2–(3,4,5-trimethoxyphenyl)-3,5-dihydro-4H-imidazol-4-one (13e)

Yellow solid, Yield (84%); m.p. 253–255 °C. ^1^HNMR (400 MHz, DMSO-d_6_) δ: 12.03 (s, 1H, exchangeable, NH), 9.50 (s, 1H, Ar-H), 7.83–7.77 (m, 1H, Ar-H), 7.53 (s, 2H, Ar-H), 7.48 (s, 1H, Ar-H), 7.20–7.05 (m, 2H, Ar-H), 4.04 (s, 3H, OCH_3_), 3.90 (s, 9H, 3 OCH_3_), 3.79 (s, 3H, OCH_3_) ppm. ^13^C NMR (100 MHz, DMSO-d_6_) δ: 164.6, 161.9, 157.1, 156.9, 154.1, 142.8, 141.9, 140.0, 128.4, 125.1, 124.1, 118.9, 110.0, 109.3, 108.0, 105.3, 60.7, 57.0, 56.2, 54.4 ppm. MS (70 eV): *m/z* (%): 449 (8.35) [M^+^]; Anal. Calcd for C_24_H_23_N_3_O_6_: C, 64.16; H, 5.16; N, 9.35. Found: C, 64.11; H, 5.12; N, 9.31.

##### 2.1.2.6. 5-[(2,7-Dimethoxyquinolin-3-yl)methylene]-2–(3,4,5-trimethoxyphenyl)-3,5-dihydro-4H-imidazol-4-one (13f)

Yellow solid, Yield (86%); m.p. 241–243 °C. ^1^HNMR (400 MHz, DMSO-d_6_) δ: 12.10 (s, 1H, exch., NH), 9.57 (s, 1H, Ar-H), 7.86 (d, *J* = 8.0 Hz, 1H, Ar-H), 7.53 (s, 2H, Ar-H), 7.23 (s, 1H, Ar-H), 7.15–7.08 (m, 2H, Ar-H), 4.07 (s, 3H, OCH_3_), 3.92 (s, 6H, 2OCH_3_), 3.79 (s, 3H, OCH_3_) ppm. ^13^C NMR (100 MHz, DMSO-d_6_) δ: 163.9, 162.6, 161. 5, 158.3, 153.3, 149.0, 140.7, 140.3, 129.9, 118.7, 118.2, 117.4, 106.3, 105.7, 104.1, 60.1, 56.3, 55.7, 54.1 ppm. MS (70 eV): *m/z* (%): 449 (5.65) [M^+^]; Anal. Calcd for C_24_H_23_N_3_O_6_: C, 64.16; H, 5.16; N, 9.35. Found: C, 64.13; H, 5.10; N, 9.29.

##### 2.1.2.7. 5-((7-Isopropoxy-2-methoxyquinolin-3-yl)methylene)-2–(3,4,5-trimethoxy phenyl)-3,5-dihydro-4H-imidazol-4-one (13g)

Yellow solid, Yield (77%); m.p. 238–240 °C. ^1^HNMR (400 MHz, DMSO-d_6_) δ: 12.19 (s, 1H, exch.,NH), 9.61 (s, 1H, Ar-H), 7.92 (d, *J* = 8.0 Hz, 1H, Ar-H), 7.58 (s, 2H, Ar-H), 7.28 (s, 1H, Ar-H), 7.17 (s, 1H, Ar-H), 7.09–7.07 (d, *J* = 8.0 Hz, 1H, Ar-H), 4.90–4.83 (m, 1H, OCH), 4.09 (s, 3H, OCH_3_), 3.94 (s, 6H, 2OCH_3_), 3.79 (s, 3H, OCH_3_), 1.37 (s, 3H, CH_3_) 1.36 (s, 3H, CH_3_) ppm. ^13^C NMR (100 MHz, DMSO*-*d_6_) δ: 164.2, 161.3, 156.7, 154.2, 153.5, 142.0, 140.9, 139.7, 128.1, 124.8, 122.4, 118.6, 109.2, 108.5, 104.3, 69.8, 60.3, 56.3, 53.9, 21.7 ppm. MS (70 eV): *m/z* (%): 477 (9.25) [M^+^]; Anal. Calcd for C_26_H_27_N_3_O_6_: C, 65.40; H, 5.70; N, 8.80. Found: C, 65.35; H, 5.66; N, 8.86.

##### 2.1.2.8. 5-[(7-(Benzyloxy)-2-methoxyquinolin-3-yl)methylene]-2–(3,4,5-trimethoxy phenyl)-3,5-dihydro-4H-imidazol-4-one (13h)

Yellow solid, Yield (73%); m.p. 259–261 °C. ^1^H NMR (400 MHz, DMSO-d_6_) δ: 12.16 (s, 1H, exch., NH), 9.62 (s, 1H, Ar-H), 7.96–7.94 (d, *J* = 8 Hz, 1H, Ar-H), 7.58–7.28 (m, 10H, ArH), 5.30 (s, 2H, OCH_2_-), 4.09 (s, 3H, OCH_3_), 3.94 (s, 6H, 2OCH_3_), 3.79 (s, 3H, OCH_3_) ppm. ^13^C NMR (400 MHz, DMSO-d_6_) δ: 163.9, 161.6, 158.5, 153.5, 148.8, 140.7, 140.3, 136.6, 130.2, 128.5, 128.0, 127.8, 118.9, 118.5, 117.8, 107.2, 105.9, 104.0, 69.7, 60.4, 56.3, 54.0 ppm. MS (70 eV): *m/z* (%): 525 (6.75) [M^+^]; Anal. Calcd for C_30_H_27_N_3_O_6_: C, 68.56; H, 5.18; N, 8.00. Found: C, 68.51; H, 5.13; N, 8.05.

### 2.2. Biochemical evaluation of activity

All biochemical assays were performed in triplicate on at least three independent occasions for the determination of mean values.

#### 2.2.1. Cell culture

The four human tumour cell lines MCF-7, HCT-116, HL-60 and HeLa used in this study were obtained from the VACSERA (Giza, Egypt) cell culture unit that were originally acquired from ATCC (Manassas, VA, USA). All the human tumour cell lines were cultured in Dulbecco’s Modified Eagle’s Medium (DMEM) with 10% foetal bovine serum, 2 mM L-glutamine and 100 µg/mL penicillin/streptomycin. Cells were maintained at 37˚C in 5% CO_2_ in a humidified incubator. All cells were sub-cultured 3 times/week by trypsinisation using TrypLE Express (1X).

#### 2.2.2. Cell viability assay

The quinoline compounds were evaluated for antiproliferative effect using the MTT viability assay of four cancer cell lines (MCF-7, HCT-116, HL-60 and HeLa) and normal breast cells MCF-10A to calculate the relative IC_50_ values for each compound. Cells were seeded in triplicate in 96-well plates at a density of 10 × 10^3^ cells/ml in a total volume of 200 µl per well. 0.1% of DMSO was used as a vehicle control. Following, the cells were treated with 2 µl test compound (from stock solutions in ethanol) to furnish the concentration range of study, 1 nM to 50 µM, and re-incubated for a further 72 h. The culture medium was then removed, and the cells washed with 100 µL phosphate buffered saline (PBS) and 50 µL MTT added, to reach a final concentration of 1 mg/mL. Cells were incubated for 2 h in darkness at 37 °C. Solubilisation was begun through the addition of 200 ml DMSO, and the cells maintained at room temperature in darkness for 20 min to ensure thorough colour diffusion before reading the absorbance. Plates were incubated for 72 h at 37 °C + 5% CO_2_. The MTT (5 mg/mL in PBS) was added and incubated for another 4 h, and the optical density was detected with a microplate reader at 570 nm. Results were expressed as percentage viability relative to vehicle control (100%). Dose response curves were plotted and IC_50_ values (concentration of drug resulting in 50% reduction in cell survival) were obtained using the commercial software package Prism (GraphPad Software, Inc., La Jolla, CA, USA). All the experiments were repeated in at least three independent experiments.

#### 2.2.3. Tubulin polymerisation assay

The assembly of purified bovine tubulin was monitored using a kit, BK006, purchased from Cytoskeleton Inc., (Denver, CO, USA). The assay was carried out in accordance with the manufacturer’s instructions using the standard assay conditions^49^. Briefly, purified (>99%) bovine brain tubulin (3 mg/mL) in a buffer consisting of 80 mM PIPES (pH 6.9), 0.5 mM EGTA, 2 mM MgCl_2_, 1 mM GTP and 10% glycerol was incubated at 37 °C in the presence of either vehicle (2% (v/v) ddH_2_O), CA-4, or the quinoline compounds. Light is scattered proportionally to the concentration of polymerised microtubules in the assay. Therefore, tubulin assembly was monitored turbidimetrically at 340 nm in a Spectramax 340 PC spectrophotometer (Molecular Devices, Sunnyvale, CA, USA). The concentration that inhibits tubulin polymerisation by 50% (IC_50_) was determined using area under the curve (AUC). The AUC of the untreated controls were considered as 100% polymerisation. The IC_50_ value for each compound was computed using GraphPad Prism Software.

#### 2.2.4. Colchicine site competitive binding assay

The affinity of compounds **12c** to colchicine binding site was determined using Colchicine Site Competitive Assay kit CytoDYNAMIX Screen15 (Cytoskeleton, Inc., Denver, CO, USA) using the standard protocol of the manufacturer to determine Ki. Biotin-labelled tubulin (0.5 µg) in 10 µL of reaction buffer was mixed with [3H]colchicine (0.08 µM, PerkinElmer, Waltham, MA) and the test compounds (positive control colchicine, negative control vinblastine, G-1, fluorescent G-1, or 2-ME) in a 96-well plate (final volume: 100 µL). After incubating for 2 h at 37 °C with gentle shaking, streptavidin-labelled yttrium SPA beads (80 µg in 20 µL reaction buffer, PerkinElmer, Waltham, MA) were added to each well and incubated for 30 min at 4 °C. The plates were then read on a scintillation counter (Packard Instrument, Topcount Microplate Reader) and the percentage of inhibition was calculated^50^^,^^51^.

#### 2.2.5. Cell cycle analysis

MCF-7 cells were seeded at a density of 1 × 10^5^ cells/well in 6-well plates and treated with CA-4 (50 nM) and compound **12c** (50 and 250 nM) for 24, 48 and 72 h. The cells were collected by trypsinisation and centrifuged at 800×*g* for 15 min. Cells were washed twice with ice-cold PBS and fixed in ice-cold 70% ethanol overnight at −20 °C. Fixed cells were centrifuged at 800×*g* for 15 min and stained with 50 µg/mL of PI, containing 50 µg/mL of DNase-free RNase A, at 37 °C for 30 min. The DNA content of cells (10,000 cells/experimental group) was analysed by flow cytometer at 488 nm using a FACSCalibur flow cytometer (BD Biosciences, San Jose, CA) and all data were recorded and analysed using the CellQuest Software (Becton-Dickinson).

#### 2.2.6. Annexin V/PI apoptotic assay

Apoptotic cell death was detected by flow cytometry using Annexin V and propidium iodide (PI). MCF-7 Cells were seeded in 6 well plated at density of 1 × 10^5^ cells/mL and treated with vehicle (0.1% (v/v) EtOH), positive control (CA-4) or compound **12c** (50 and 250 nM) for 24, 48 and 72 h. Cells were then harvested and prepared for flow cytometric analysis. Cells were washed in 1X binding buffer (20X binding buffer: 0.1 M HEPES, pH 7.4; 1.4 M NaCl; 25 mM CaCl_2_ diluted in dH_2_O) and incubated in the dark for 30 min on ice in Annexin V-containing binding buffer [1:100]. Cells were then washed once in binding buffer and then re-suspended in PI-containing binding buffer [1:1000]. Samples were analysed immediately using the BD accuri flow cytometer and prism software for analysis the data. Four populations are produced during the assay Annexin V and PI negative (Q4, healthy cells), Annexin V positive and PI negative (Q3, early apoptosis), Annexin V and PI positive (Q2, late apoptosis) and Annexin V negative and PI positive (Q1, necrosis).

#### 2.2.7. Evaluation of expression levels of anti-apoptotic proteins bcl-2, pro-apoptotic proteins bax and caspase 9

The level of the anti-apoptotic marker and apoptotic marker BAX were assessed using Bcl-2 Elisa kit and human Bax ELISA Kit purchased from Zymed laboratories, invitrogen and Cloud-Clone Crop. (Katy, TX, USA), respectively, following the manufacturer’s instructions. Briefly, Treated MCF-7 cell lysate with 250 nM of compound **12c** were prepared, and equal amount of cell lysates were loaded and propped with specific antibodies. The samples were measured and analysed at 450 nm in ROBONEK P2000 ELISA reader^52^. *In Vitro* Caspase-9 Activation Assay was performed using human active caspase-9 Invitrogen EIA kit according to the manufacturer’s instructions. Compound **12c** at concentrations of 50 and 250 nM and CA-4 (50 nM) were prepared in dH_2_O up to a final volume of 50 µL/well followed by addition of 5 µL of active caspase-9. Following, the cells were mixed and 50 µL of the Master Mix was added to each well and allowed to react at 37 °C for 1 h. The fluorescence intensity of the test samples was recorded and analysed in a fluorescence plate reader at 400 nm excitation and 505 nm emissions. All experiments were conducted in triplicates.

#### 2.2.8. Colony formation assay

MCF-7 cells (600 cells per well) were seeded in 6-well plates and incubated for 24 h before being then treated with different doses of the compound **12c** (50 and 250 nM) for 14 days. Following, the cells were washed with PBS twice and subsequently fixed with 4% paraformaldehyde and stained with 0.05% crystal violet for 30 min. Finally, cells were visualised using an inverted microscope.

#### 2.2.9. Wound healing assay

MCF-7 were grown in 6-well plates for 24 h, and scratches were made using pipette tip and washed with PBS to remove non-adherent cell debris. Subsequently, the cells were treated with different concentrations of **12c** for 24 h. The migrations across the wound area were photographed under a phase contrast microscopy.

#### 2.2.10. Measurement of mitochondrial depolarisation effect (Δψ_mt_) and ROS levels in cells

Mitochondrial membrane potential (Δψ_mt_) was measured by flow cytometry with DiOC2(3) staining and additional labelling with an annexin V conjugate. After treatment with compound **12c** (50 and 250 nM) and CA-4 (50 nM), cells were stained with DiOC2(3) dye for 30 min in the incubator, then harvested and washed with PBS. DiOC2(3)-stained cells were resuspended with 1X annexin binding buffer, followed by addition of annexin V conjugate and incubated at 37 °C for 15 min. The data from the flow cytometry were analysed by Cell Quest software. Production of intracellular reactive oxygen species (ROS) was measured using 2,7–dichlorofluorescin diacetate (H_2_-DCFDA) dye. MCF-7 cells were seeded and treated either with vehicle (0.1% DMSO) or with compound **12c** (50 and 250 nM) or CA-4 (50 nM) for 6, 12 and 24 h. H_2_O_2_ was used as a positive control. The amount of ROS generated was estimated after 2 h of selected compound treatment. The cells were collected by centrifugation and washed twice with PBS. Cells were then incubated with DCFDA dye (25 µM) in dark at 37 °C for 1 h. Fluorescence spectra (510 − 600 nm) were monitored using an excitation wavelength of 488 nm^53^^,^^54^.

## 3. Results and discussion

### 3.1. Design and chemistry

The natural product, Combretastatin A-4 (CA-4; Figure 1) exhibits significant antiproliferative activities against several tumour cells by binding to the colchicine site of tubulin to inhibit the protein polymerisation^20^. However, the *cis* double bond of CA-4 has a propensity to isomerise into the inactive *trans* configuration, leading to reduction in the molecule’s pharmacologic activity. Several structural modifications of the CA-4 pharmacophore have subsequently been undertaken to overcome this disadvantage, e.g. replacing the *cis* double bond with a heterocycle, oxadiazole, isoxazole and imidazole, resulting in compounds, such as **1**, **2** and **3** respectively (Figure 1)^27–32^. In this work, we undertook a rational design approach of introducing chalcone system (ring C) in the form of either oxazolone or imidazolone between the two rings A and B, as well as isosterically replace ring B with quinolone. Specifically, the rigidity of the molecules was increased by introducing 1,3-oxazol-5-ones and 1,3-imidazol-4-ones to the *cis-*olefinic bond of CA-4, which we anticipate would create a desirable conformational and configurational restriction to prevent isomerisation of CA-4 into the inactive *trans*-isomer, as well as improve on the anticancer activities of these compounds since chalcones are well known for their anticancer properties^55^^,^^56^. The second design step involves varying the electronic substituents effect on the quinolyl moiety (ring B), while maintaining the natural active compound 3,4,5-trimethoxyphenyl moiety, which we anticipate will increase the potency of these compounds.

**Scheme 1.**
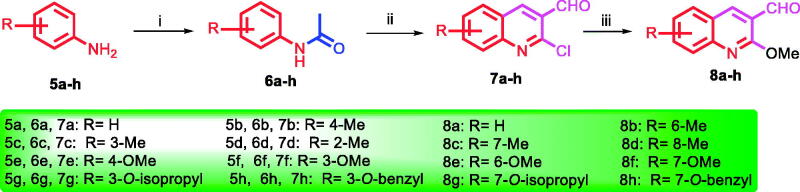
Synthetic route for preparation of aldehydes **8a–h**. Reagents and conditions: (i) Ac_2_O, AcOH, 0 °C,1h; (ii) DMF, POCl_3_, 70–90 °C, 18h; (iii) CH_3_ONa, MeOH, 40 °C, 3–6h.

**Scheme 2.**
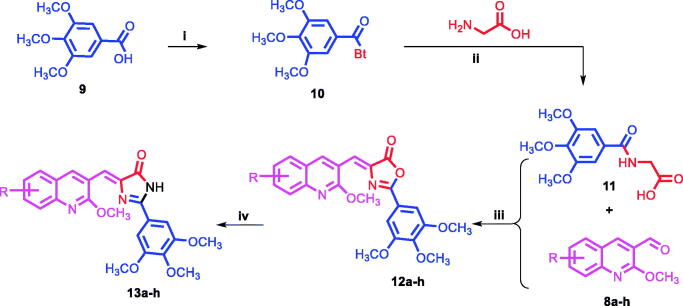
Synthetic route for preparation of Quinoline targets **12a–h** and **13a–h**. Reagents and conditions: (i) SOCl_2_,DCM, r.t; (ii) TEA, MeCN,H_2_O, r.t; (iii) AcONa, AC_2_O, 80 °C, 2h; (iv) NH_4_OH, EtOH, reflux, 18h.

The syntheses of the proposed quinoline compounds **12a–h** (oxazolones) and **13a–h (**imidazolones) (Table 1) are shown in Schemes 1 and 2 and involve two core structural components: (i) 2-methoxyquinolyl-3-carbaldehyde nucleus **8a–h**, and (ii) 3,4,5-trimethoxyphenyl moiety **11**. A concise (three-step) synthesis was used for the synthesis of the first core structure, 2-methoxyquinoline-3-carbaldehyde derivatives **8a–h,** as shown in Scheme 1. The synthesis was initiated with acetylation of the starting aniline derivatives **5a–h** using acetic anhydride and glacial acetic acid at 0 °C. The produced amides **6a–h** were subjected to Vilsmeier–Haack reaction to give the corresponding quinoline-3-aldehyde derivatives **7a–h**. Addition of methoxy substituent to **7a–h** to give **8a–h** was achieved through the use of sodium methoxide at 40 °C in methanol^47^^,^^48^. The synthesis of the second core 3,4,5-trimethoxyphenyl moiety **11** started with acylation of the acid **9** under highly acidic condition using SOCl_2_ to give acyl benzotriazole **10** (Scheme 2)^57^^,^^58^. Following, treatment of the acyl benzotriazole **10** with glycine in aqueous acetonitrile gave the acyl glycine **11**. Condensation of **11** with the appropriate quinoline aldehydes **8a–h** in the presence of acetic anhydride and catalytic amount of sodium acetate resulted in the formation of the oxazolones **12a–h**. Aminolysis of **12a–h** via condensation reaction with ammonia led to the formation of the imidazolones **13a–h**. It seems the nucleophilic ammonia attacks the carbonyl group of the oxazolone ring, followed by immediate intramolecular condensation and cyclisation to give the imidazolones **13a–h**. In summary, two classes of compounds, **12a–h** (oxazolones) and **13a–h** (imidazolones) were synthesised and used for further functional and biological studies.

**Table 1.** Antiproliferative activity of quinoline analogues against human cancer cell lines (IC_50_ [µM]). 
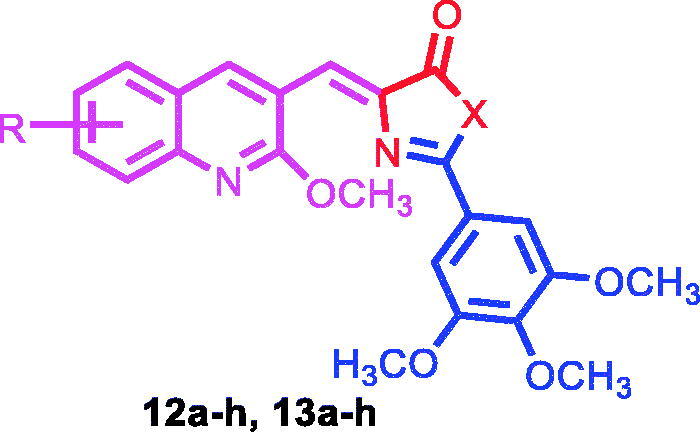

Compound number	X	R	IC_50_ value (µM)^a^			
			HL-60	MCF-7	HCT-116	HeLa
**12a**	O	H	3.309 ± 0.022	1.712 ± 0.040	2.012 ± 0.080	2.113 ± 0.034
**12b**	O	6-CH_3_	0.197 ± 0.041	0.132 ± 0.013	0.184 ± 0.007	0.117 ± 0.085
**12c**	O	7-CH_3_	0.019 ± 0.059	0.010 ± 0.003	0.022 ± 0.001	0.042 ± 0.001
**12d**	O	8-CH_3_	0.268 ± 0.093	0.154 ± 0.071	0.191 ± 0.001	0.153 ± 0.083
**12e**	O	6-OCH_3_	0.068 ± 0.034	0.056 ± 0.027	0.031 ± 0.001	0.010 ± 0.004
**12f**	O	7-OCH_3_	0.352 ± 0.021	0.052 ± 0.0021	0.066 ± 0.005	0.138 ± 0.026
**12g**	O	7-OCH(CH_3_)_2_	1.822 ± 0.640	1.507 ± 0.220	7.880 ± 0.300	1.747 ± 0.500
**12h**	O	7-OCH_2_Ph	4.660 ± 0.260	1.932 ± 0.612	1.563 ± 0.720	1.054 ± 0.840
**13a**	NH	H	1.561 ± 0.055	1.033 ± 0.055	8.21 ± 0.0077	1.409 ± 0.096
**13b**	NH	6-CH_3_	0.240 ± 0.012	0.063 ± 0.0011	0.173 ± 0.025	0.188 ± 0.013
**13c**	NH	7-CH_3_	0.661 ± 0.026	0.223 ± 0.056	0.284 ± 0.083	0.733 ± 0.091
**13d**	NH	8-CH_3_	0.096 ± 0.006	0.137 ± 0.023	0.109 ± 0.011	0.126 ± 0.044
**13e**	NH	6-OCH_3_	0.272 ± 0.050	0.042 ± 0.0025	0.085 ± 0.003	0.062 ± 0.003
**13f**	NH	7-OCH_3_	0.210 ± 0.098	0.092 ± 0.0062	0.187 ± 0.009	0.101 ± 0.090
**13g**	NH	7-OCH(CH_3_)_2_	5.114 ± 0.410	5.562 ± 0.133	1.620 ± 0.950	4.439 ± 0.600
**13h**	NH	7-OCH_2_Ph	4.931 ± 0.260	1.695 ± 0.821	2.610 ± 0.430	2.070 ± 0.390
**CA-4**	–	–	0.076 ± 0.004	0.019 ± 0.004	0.026 ± 0.001	0.064 ± 0.004

^a^IC_50_ values are half maximal inhibitory concentrations required to block the growth stimulation of cells. Values represent the mean for three experiments performed in triplicate.

### 3.2. Biological results and discussion

#### 3.2.1. *In vitro* antiproliferative activities

All the synthesised compounds (with CA-4 as a positive reference) were evaluated for their antiproliferative activities using MTT assay with four different cancer cell lines – MCF-7 breast adenocarcinoma, HL-60 leukaemia, HCT-116 colorectal carcinoma, and HeLa cervical adenocarcinoma. As shown in Table 1, most of the compounds demonstrated moderate to highly potent antiproliferative activities. In the oxazolone analogues (**12a**–**h**), compound **12a** without any substituent on the quinoline ring was the least active when compared with quinoline ring substituted compounds. The relative position of the substituent on the quinoline ring also seemed to be critical for antiproliferative activity. Compound **12c** with methyl group at the 7-position ring displayed impressive non-selective potency in nanomolar range against HL-60, MCF-7, HCT-116 and HeLa cell lines with IC_50_ of 0.019, 0.010, 0.022 and 0.042 µM, which compared to 0.076, 0.019. 0.026 and 0.064 µM for CA-4, respectively. In contrast, both the 6-CH_3_ analog (**12b**) and 8-CH_3_ analog (**12d**) were 3- to 15-fold less active than **12c**. The nature of the substituents on the quinoline ring of the oxazolone compounds was also found to significantly influence the biological activity. For example, replacement of the methyl group in **12b** and **12c** with a stronger electron-releasing methoxy group yielded compounds **12e** and **12f**, respectively, which resulted in better antiproliferative activities. The methoxy-containing compound **12e** was 2.7- to 13-fold more active than the methyl-containing compound **12b** with the four cancer cell lines (IC_50_ of 0.068, 0.056, 0.031 and 0.010 µM in HL-60, MCF-7, HCT-116 and HeLa cancer cell lines, respectively). Compound **12f** had a similar effect as **12c** against MCF-7 and HCT-116 cells (0.052 and 0.066 µM, respectively), but with reduction in activity against the other two cell lines, HL-60 and HeLa (0.352 and 0.138 µM, respectively). Introducing larger substituents at the quinoline ring as in **12g** (7-*tert*-butyl) and **12h** (7-benzyloxy) led to a dramatic decrease in activity compared to their corresponding analog **12f** (7-methoxy). Summarily, adding smaller and/or polar groups to the quinoline ring of the oxazolone resulted in significant improvement in the antiproliferative activity.

The imidazolones (Compound **13a**–**h)** also resulted in impressive antiproliferative activity with IC_50_ values ranging from 0.04–8.21 µM in all four cell lines. In general, the imidazolones showed similar antiproliferative activities as the oxazolones (Table 1), which could be due to similar electronic effects of the oxazolone and imidazolone rings. Like the oxazolone, lack of substituent on the quinolone ring as in compound **13a** led to reduction in activity, with IC_50_ values of more than 1 µM in all four cell lines, similar to the results obtained with the oxazolone derivative **12a**. Methyl substitution on the quinoline ring, e.g. 6-CH_3_ **13b**, 7-CH_3_ **13c** and 8-CH_3_ **13d** led to potent activity in submicromolar range in all four cancer cell lines.

The position of the methoxy substituent on the quinoline heterocycle also influenced the antiproliferative activity of the compounds against the cancer cell lines. For example, the antiproliferative activity of 6-methoxy-substituted **13e** was better than its analog 7-methoxy-substituted **13f** against MCF-7, HCT-116 and HeLa cells with IC_50_ values of 0.042, 0.085 and 0.062 µM, which compare to 0.092, 0.187 and 0.101 µM for **13f**, respectively. However, in HL-60, **13e** exhibited less antiproliferative activity with IC_50_ value of 0.272 µM. In a similar trend as the oxazolone derivatives, bulky substituents on the quinoline ring **13g** (7-*tert*-butyl) and **13h** (7-benzyloxy) resulted in drastic decrease in activity in all four cancer cell lines with 14- to 125-fold loss in potency compared to their corresponding **13f** (7-methoxy containing) compound.

In summary, both oxazolone and imidazolone compounds displayed potent antiproliferative effects, strengthening our hypothesis that nitrogen-containing heterocycles, such as quinoline, are beneficial surrogates for the ring B of CA-4. The different biological activities of the compounds are likely the result of differences in their mode of interaction with the colchicine binding site. Due to its excellent antiproliferative activity, compound **12c** was studied in more details as described below.

#### 3.2.2. *In vitro* inhibition of tubulin polymerisation and colchicine binding

Trimethoxyphenyl (TMP) containing stilbenoid derived compounds, such as colchicine, resveratrol and CA-4 bind to tubulin at the colchicine binding site, resulting in inhibition of microtubule polymerisation^59^^,^^60^. To confirm whether the quinoline compounds similarly target the tubulin-microtubule system, representative quinoline compounds, including four oxazolone analogues (**12a**, **12c**, **12e** and **12g**) and two imidazolone analogues (**13c** and **13e**), as well as the reference compound CA-4, were evaluated for their antitubulin polymerisation activities and the results presented in Table 2. The methyl and methoxy substituted oxazolone compounds **12c** and **12e,** respectively strongly inhibited tubulin assembly with IC_50_ of 1.21 and 2.26 µM, respectively compared to that of CA-4 (IC_50_ of 2.17 µM), while the unsubstituted analogue **12a** (IC_50_ of 13.98 µM) and *tert-*butyl analog **12g** (IC_50_ of 8.23 µM) were 6- and 4-fold less active than CA-4. The imidazolone compound **13e** with IC_50_ of 1.48 µM also showed very potent tubulin polymerisation inhibition compared to CA-4. The methyl analogue **13c** was inactive in the tubulin polymerisation assay (IC_50_ of 20.29 µM), and is 16-fold less active compared to its corresponding oxazolone derivative **12c**, which is in agreements with the poor cell growth inhibitory activity of **13c** compared to **12c**.

**Table 2.** Inhibition of Tubulin Polymerisation and Colchicine Binding by quinoline compounds and CA-4.

Compound number	Tubulin assembly^a^	Colchicine binding^b^	
	IC_50_ (µM)	%± SD	
		1 µM drug	5 µM drug
**12a**	13.98	nd	nd
**12c**	1.21	79 ± 2	87 ± 1
**12e**	2.26	nd	nd
**12g**	8.23	nd	nd
**13c**	20.29	nd	nd
**13e**	1.48	nd	nd
**CA-4**	2.17	86 ± 0.9	97 ± 2

^a^Inhibition of tubulin polymerisation. Tubulin was at 10 µM.

^b^Inhibition of [^3^H] colchicine binding. Tubulin and colchicine were at 1 and 5 µM concentrations, respectively.

Compound **12c** was also examined at two different concentrations (1 and 5 µM) for its ability to compete with colchicine for binding to tubulin using a [^3^H] colchicine binding assay. Compound **12c** strongly inhibited colchicine binding to tubulin by 79% and 87% at 1 and 5 µM respectively, which compares with 86% and 97% inhibition by CA-4, respectively. These results suggest that compound **12c** is involved in tubulin polymerisation inhibition through the colchicine-binding site.

#### 3.2.3. Cell cycle analysis

**Figure 3.**
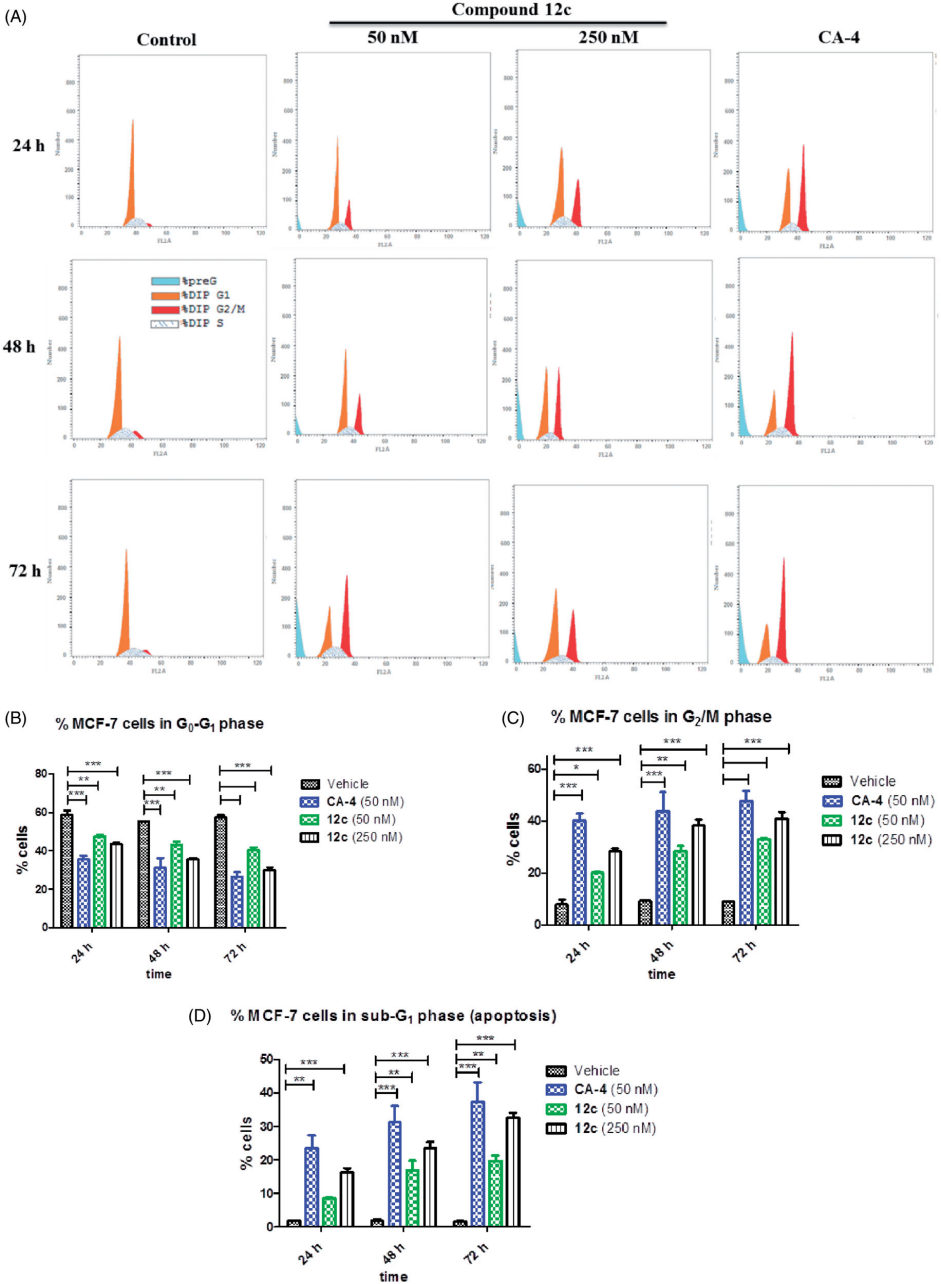
(A) Effect of compound **12c** on the cell cycle and apoptosis in MCF-7 cells. Cells were treated with either vehicle [0.1% ethanol (v/v)], CA-4 (50 nM), **12c** (50 nM and 250 nM) for 24 h, 48 h and 72 h. Cells were then fixed, stained with PI, and analysed by flow cytometry. Cell cycle analysis was performed on histograms of gated counts per DNA area (FL2-A). The number of cells with (B) 4 N (G_2_/M), (C) 2 N(G_0_G_1_), and (D) <2 N (sub-G_1_) DNA content was determined with CellQuest software. Values represent the mean ± SEM for three independent experiments. Statistical analysis was performed using two-way ANOVA (*p < 0.05; **p < 0.01; ***p < 0.001).

Induction of cell cycle arrest at G_2_/M phase is strongly accompanied with tubulin polymerisation inhibition. It is well established that CA-4 arrests cell cycle at G_2_M phase^61–63^. To further gain insight into compound **12c** potent antiproliferative activity, cell cycle analysis of MCF-7 cells was performed at two concentrations of 50 nM and 250 nM and at different time points of 0, 24, 48 and 72 h. Figure 3(A) clearly demonstrates that **12c** caused a significant arrest in G_2_/M phase and apoptosis in a dose- and time-dependent manner. After 48 h, the percentage of G_2_/M phase arrested cells were 28.4% and 38.3% at 50 nM and 250 nM, respectively compared to 9.2% of untreated cells (Figure 3(B)). Moreover, there was an increase in the number of cells in G_2_/M phase after 72 h (33.0% and 40.8% at 50 nM and 250 nM, respectively) with a concomitant decrease of cells in G_0_/G_1_ phase (40.3% and 29.8% at 50 nM and 250 nM, respectively) compared to the control (57.3%). In a comparable finding, CA-4 (50 nM) also significantly arrested G_2_/M phase at 24, 48 and 72 h (40.3%, 43.8% and 47.7%, respectively). Accordingly, a concomitant decrease of MCF-7 cells was detected in G0 phase (Figure 3(C)). Furthermore, compound **12c** induced a gradual increase in apoptosis (16.2%, 23.4% and 32.7%) at 250 nM as the proportion of cells in the sub-G1 phase increased at 24, 48 and 72 h, respectively compared to untreated cells (1.5%) (Figure 3(D)). Similarly, 23.5%, 31.1% and 37.4% increase in apoptosis was observed for CA-4 at 24, 48 and 72 h, respectively. These findings are in agreement with previously reported for a series of related quinoline analogues, which significantly induced apoptosis and G_2_/M cycle arrest in MCF-7 cells^23^^,^^42^^,^^46^^,^^64^.

#### 3.2.4. Cell apoptosis

**Figure 4.**
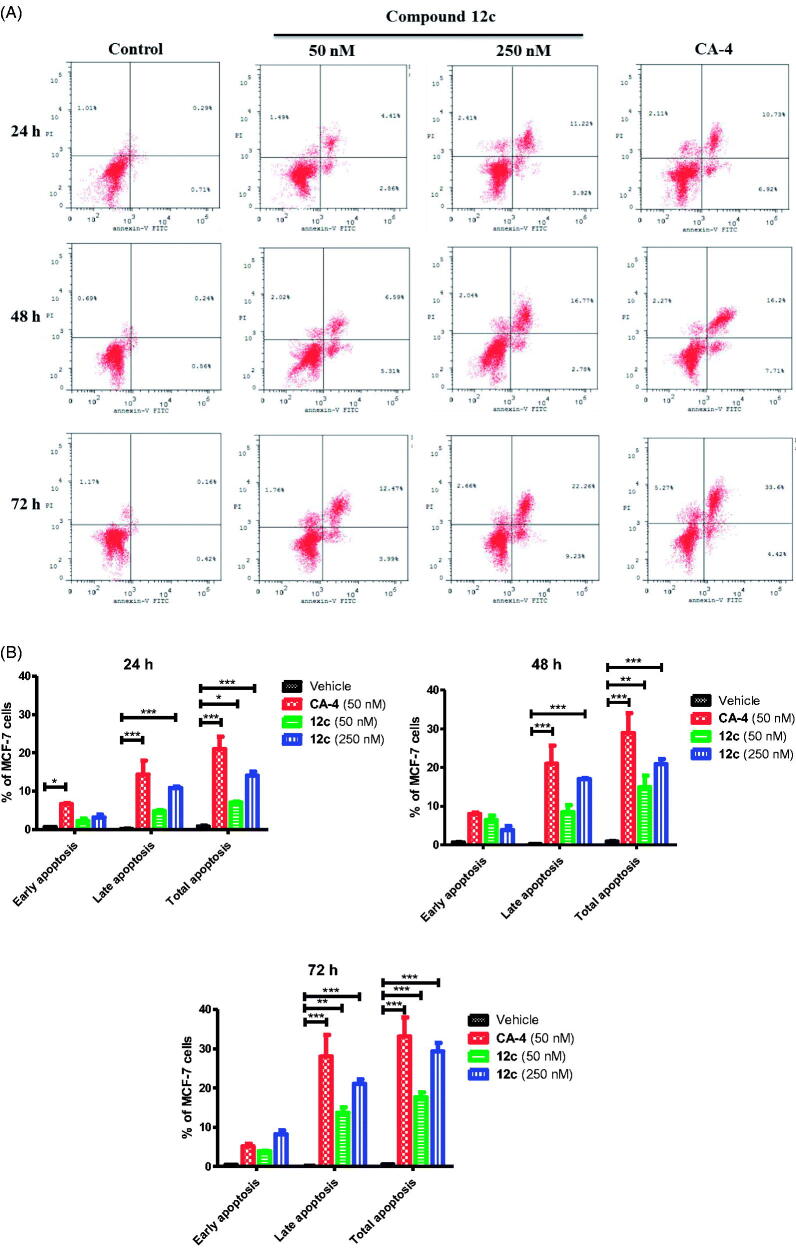
(A) Effect of compound **12c** at different time points on apoptosis in MCF-7 cells analysed by flow cytometry after double staining of the cells with Annexin-V-FITC and PI. MCF-7 cells treated with 50 and 250 nM of compound **12c** and 50 nM of CA-4 for 24 h, 48 h and 72 h and collected and processed for analysis. (B) Quantitative analysis of apoptosis. Values represent the mean ± SEM for three independent experiments. Statistical analysis was performed using two-way ANOVA (*p < 0.05** and *p* < 0.01; ***p < 0.001).

We investigated whether cell death induced by compound **12c** treatment was related to apoptosis using the Annexin-V/PI double staining flow cytometric assay (Figure 4(A,B)). MCF-7 cells were treated with three different concentrations (0, 50 and 250 nM) of compound **12c** at different time points (24, 48 and 72 h). Compound **12c** caused a significant accumulation of annexine-V positive cells and induced both early and late apoptosis in a dose- and time-dependent manner compared to the untreated cells. As shown in Figure 4(B), when the cells were treated with **12c (**0 and 250 nM) or CA-4 (50 nM) for 48 h, the percentage of Annexin V-staining positive cells significantly increased from 1% in untreated cells to 15%, 21% and 29% respectively. The percentage of early and late apoptotic cells in the presence of **12c** increased after 72 h to 17.6% and 29.3% at 1 and 5 µM respectively when compared to the untreated cells (2%). Based on the cell cycle arrest and apoptosis findings (Figure 3(B–D)), it appears that compound **12c** could efficiently induce apoptosis cell death in MCF-7 cells in a dose- and time-dependent manner.

#### 3.2.5. Assessment of toxicity to non-tumorigenic human cells

**Figure 5.**
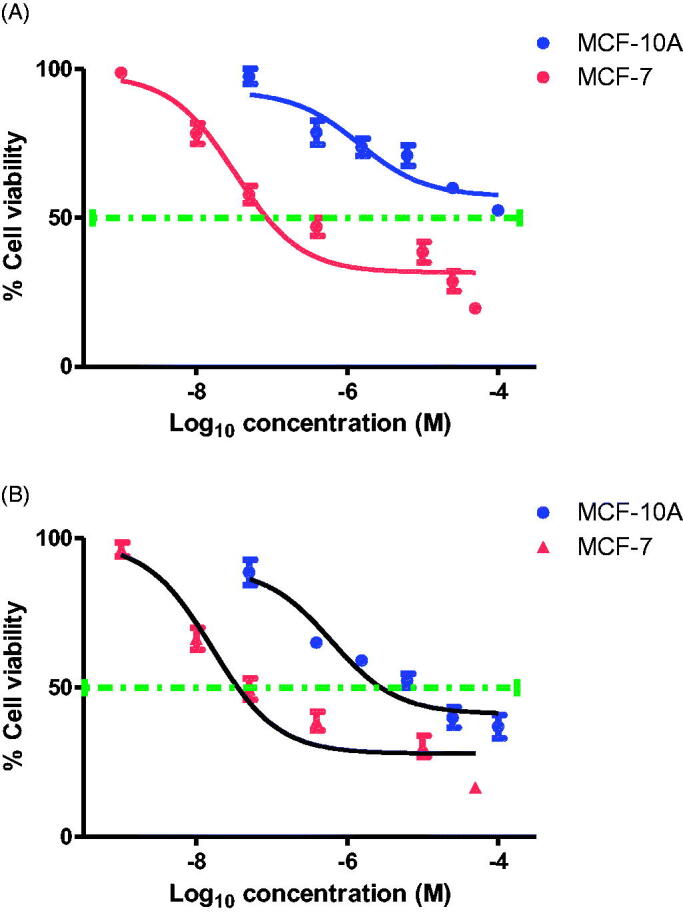
Dose response curve for (A) Compound **12c** and (B) CA-4 on the proliferation of breast cancer MCF-7 and normal breast MCF-10A cells. Cells were grown in 96-well plates and treated with serial concentrations of compound **12c or** CA-4 for 72 h. Cell viability was expressed as percentage of vehicle control [ethanol 1% (v/v)] treated cells and was measured by MTT assay (average of three independent experiments).

To assess the cytotoxicity and selectivity of **12c** towards cancer cells, normal epithelial breast MCF-10A cell viability study was carried out. As shown in Figure 5(A), the IC_50_ value of **12c** was more than 50 µM in MCF-10A cells, which was significantly higher than the IC_50_ values of 19, 10, 22 and 42 nM in MCF-7, HL-60, HCT-116 and HeLa cancer cell lines, respectively. Remarkably, **12c** was found to be less toxic in normal MCF-10A (IC_50_ >50 µM) when compared to CA-4 (IC_50_ = 6.1 µM) (Figure 5(B)), suggesting **12c** to have better selective toxicity against cancer cells.

#### 3.2.6. Expression of the apoptotic proteins in MCF-7 cell lines

**Figure 6.**
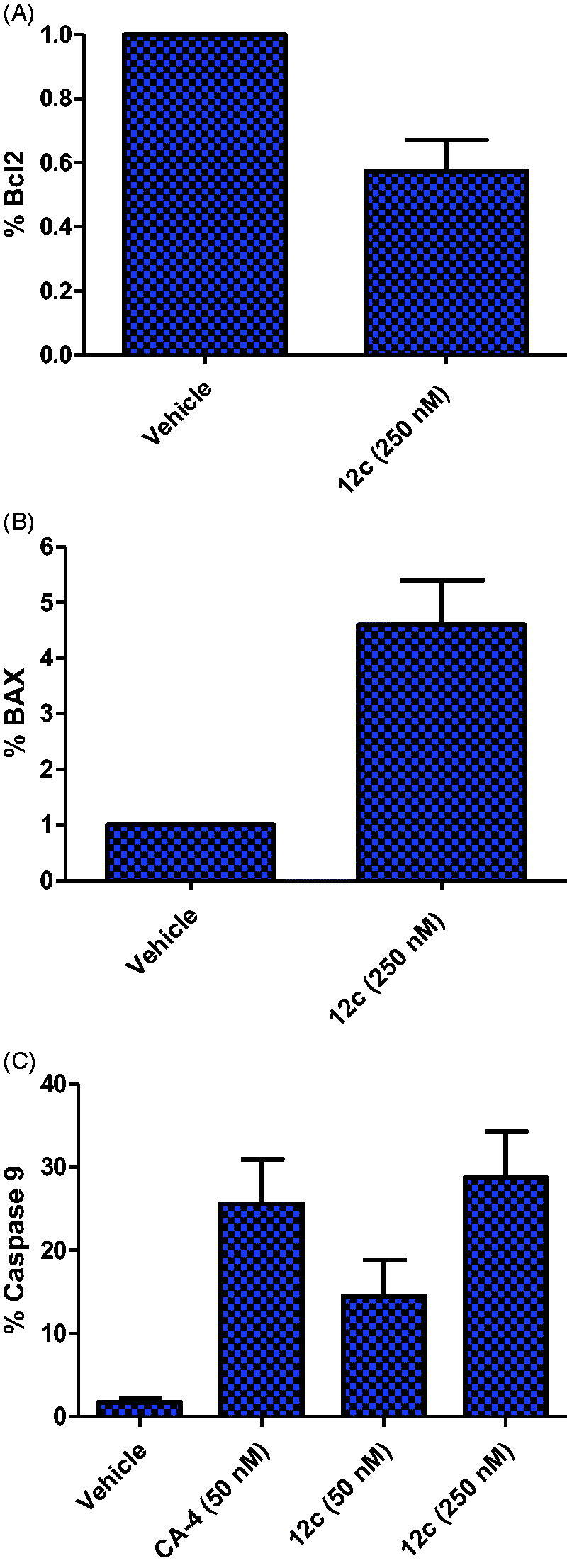
Effect of compound **12c** on the expression (A) anti-apoptotic protein Bcl2, (B) pro-apoptotic protein BAX and (C) Caspase 9 in MCF-7 cells.

The previous data clearly demonstrate that **12c** is an effective anti-mitotic quinoline compound in MCF-7 cell lines. Herein, the effect of **12c** on the expression of apoptosis pathway markers, Bcl-2 anti-apoptotic protein and Bax pro-apoptotic protein was investigated. MCF-7 cells treated with **12c** at 250 nM for 48 h decreased the expression level of the anti-apoptotic protein Bcl-2, and correspondingly up-regulated the expression of the pro-apoptotic protein Bax (Figure 6(A,B)).

Activation of caspases initiates apoptosis, and in particular caspase-9 is considered an important effector caspase responsible for programmed cell death apoptosis activated by CA-4^61^^,^^65^^,^^66^. The amount of activated caspase-9 was examined in MCF-7 cells treated with **12c**. As observed from Figure 6(C), compound **12c** at 50 and 250 nM produced about 8- and 16-fold increases in caspase-9 activation respectively when compared to 14-fold for CA-4 (50 nM). This finding confirms that compound **12c** like CA-4 enhanced the rate of apoptosis in MCF-7 cell through caspase-9 activation.

#### 3.2.7. Inhibition of colony formation

**Figure 7.**
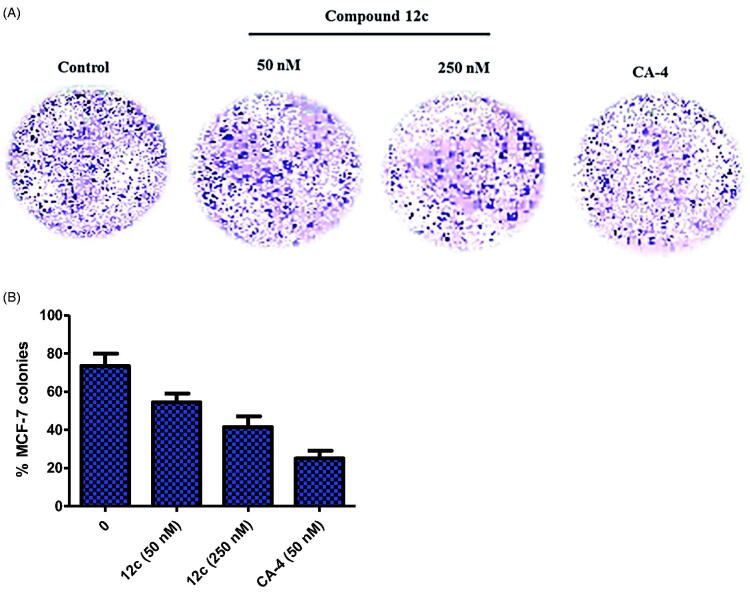
(A) Inhibition of colony formation in MCF-7 cells by 50 and 250 nM of compound **12c** and 50 nM of CA-4 for 48 h. (B) Quantitative analysis of colony formation. .

Colony formation assay is one of the effective techniques for the determination of long-term cell proliferation upon anticancer drug exposure. The inhibitory potential of **12c** on MCF-7 cells colony formation is displayed in Figure 7. Compound **12c** suppressed the clonogenic formation potential of MCF-7 cells in a dose dependent manner when compared to CA-4.

#### 3.2.8. Wound healing assay

**Figure 8.**
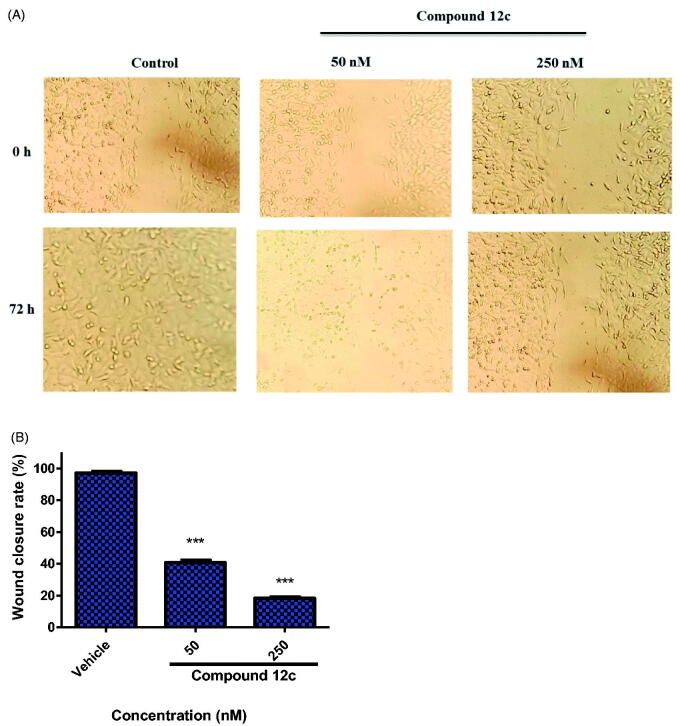
(A) Inhibition of the migration of MCF-7 cells treated with compound **12c** for 48 h in the wound healing assay. (B) Quantitative analysis of wound closure rate and was calculated as mean ± SEM for three independent experiments. Statistical analysis was performed using one-way ANOVA-Bonferroni post-hoc test (***p < 0.001).

Migration and motility of cancer cells are considered as critical factors in tumour progression and metastasis^67^^,^^68^. In order to investigate the effect of compound **12c** on the migration of MCF-7, wound healing assay was performed. As illustrated in Figure 8(A,B), the untreated cells migrated to the scraped area while in **12c**-treated wells, cell migration was significantly inhibited in a dose-dependent manner. This significant difference in the wound area confirms that **12c** suppressed MCF-7 cell migration, an important event in tumour metastasis.

#### 3.2.9. Mitochondrial membrane potential

**Figure 9.**
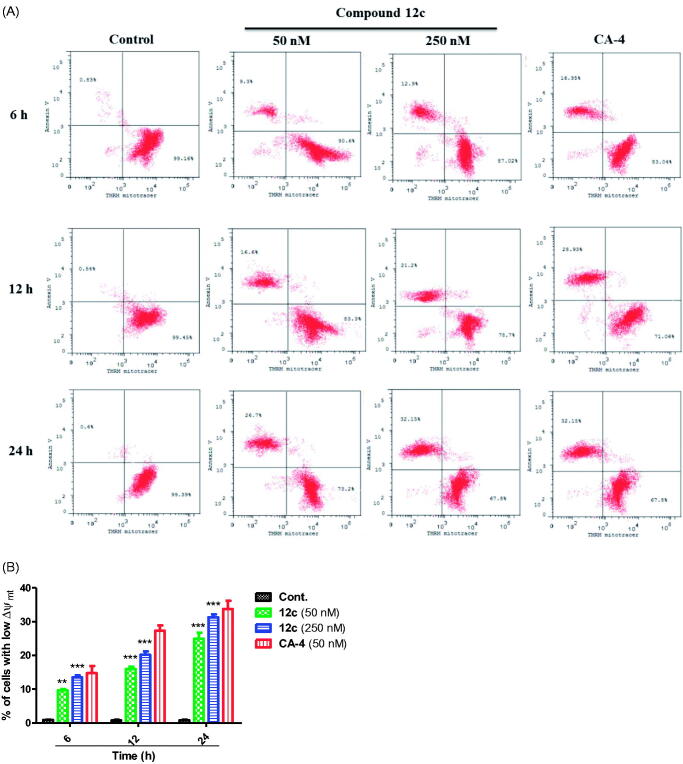
Assessment of mitochondrial membrane potential (Δψ_mt_) after treatment of MCF-7 cells with **12c**. Cells were treated with indicated concentration of compound **12c** for 6, 12 and 24 h and then stained with fluorescent DiOC2(3) for analysis of mitochondrial potential. Cells were then analysed by flow cytometry as described in the experimental section. Data are presented as mean ± SEM of three independent experiments. Statistical analysis was performed using two-way ANOVA (**p < 0.01; ***p < 0.001).

Mitochondria membrane potential plays a crucial role in the propagation of apoptosis. Specifically, loss of mitochondrial membrane potential Δψ_mt_ (MMP) is characteristic of early stage of apoptosis^69–71^. To confirm whether compound **12c** could decrease the MMP of MCF-7 cancer cells, MMP was monitored by the fluorescence of the dye DiOC2(3). MCF-7 cells treated with **12c** at 50 and 250 nM exhibited significant decrease in MMP in a dose- and time-dependant manner (Figure 9(A)). This depletion in MMP was associated with an increase of annexin-V positive early apoptotic cells. Maximum decrease in MCF-7 MMP was detected after 24 h treatment with **12c** in which the percentage of apoptotic cells increased from 1.1% to 24.9% and 31.3% at 50 and 250 nM, respectively (Figure 9(B)). This indicates that compound **12c** induces mitochondrial dysfunction in MCF-7, which eventually triggered apoptotic cell death. These results are in agreement with previously reported CA-4 analogues study that were shown to cause apoptosis through the mitochondrial pathway^68^^,^^72^^,^^73^.

#### 3.2.10. Intracellular reactive oxygen species (ROS) production

**Figure 10.**
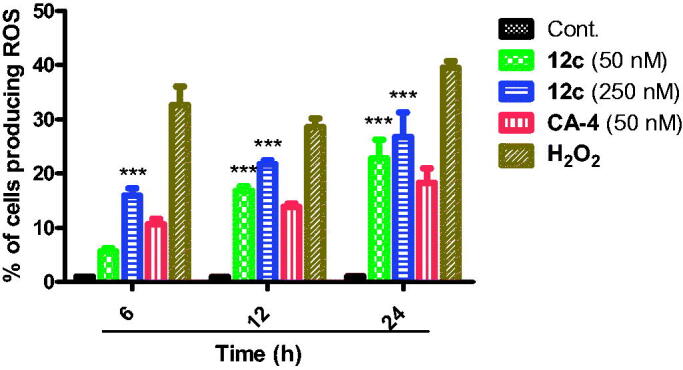
Effect of compound **12c** at different time points on ROS production in MCF-7 cells. MCF-7 cells treated without and with 50 and 250 nM of compound **12c** and 50 nM of CA-4 and 100 µM H_2_O_2_ for 6 h, 12 h and 24 h. Cells were incubated with DCFDA (25 µM) dye for 1 h at 37 °C in dark. Fluorescence intensity per cell at 525 nm was calculated as mean ± SEM for three independent experiments. Statistical analysis was performed using two-way ANOVA (***p < 0.001).

The dissipation of mitochondrial potential is strongly associated with mitochondrial production of reactive oxygen species (ROS)^71^^,^^73^. The production of ROS after **12c** treatment at 50 and 250 nM, as well as CA-4 (50 nM) with hydrogen peroxide H_2_O_2_ was followed with 2,7–dichlorofluorescin diacetate (H_2_-DCFDA). As shown in Figure 10, after 24 h of **12c** treatment, the levels of ROS in MCF-7 cells were 22.7 and 26.6% at 50 and 250 nM, respectively. The level in untreated MCF-7 cells was 1.0%, while it increased only to 18.3% in CA-4-treated cells. This result along with the significant loss of mitochondrial membrane potential above clearly suggests that compound **12c** induced apoptosis via the mitochondrial pathway.

## 4. Conclusion

In this study, we designed, synthesised and evaluated two classes of novel quinoline compounds combretastatin A-4 derivatives as potential inhibitors of tubulin polymerisation. Several other studies have also reported derivatisation of the CA-4 pharmacophore with varying success^27–32^. Unlike the previous compounds, we for the first time introduced a chalcone system, including oxazolones and imidazolones to the *cis* bond of CA-4 to give more rigidity to the required active conformation. The chalcone system is well known for its anticancer activities. Our design also kept the essential natural trimethoxyphenyl pharmacophore (found in CA-4), while varying the electronic substituents effect on the quinolyl moiety (ring B) that were expected to enhance the potency of the compounds. Most of the compounds showed significant and, in some instances, comparable antiproliferative activities against different cancer cell lines as the previously studied combretastatin A-4 compound, CA-4. One of the most promising compound **12c** showed potent anti-proliferative activities against HL-60, MCF-7, HCT-116 and HeLA cancer cell lines with IC_50_ values of 0.019, 0.010, 0.022 and 0.042 µM, respectively, and simultaneously low cytotoxicity towards MCF-10A non-cancer cells. The microtubule polymerisation inhibitory effect of **12c** was confirmed with an *in vitro* tubulin polymerisation and colchicine inhibition assays. Compound **12c** effectively block the G_2_/M phase at the cell cycle and induce MCF-7 cell apoptosis together with significant change of Bax/Bcl expression ratio indicating involvement of mitochondrial apoptosis pathway. Further cellular mechanistic studies confirmed that **12c** inhibited MCF-7 cell migration and colony formation. In conclusion, these results highlight our novel quinoline compounds and particularly **12c** as promising anti-tubulin agent for the treatment of MCF-7 breast cancer cells. Moreover, the results point to a direction for rational development of potent tubulin polymerisation inhibitors for the treatment of cancer.
